# Supplementary material for: Zinc-dependent regulation of zinc import and export genes by Zur
Source: Nat Commun. 2017 Jun 9;8:15812. doi: 10.1038/ncomms15812 (PMC5472717; doi:10.1038/ncomms15812)
Supplement: Supplementary Information — Supplementary Table, Supplementary Figures and Supplementary References [file ncomms15812-s1.pdf]

Supplementary Table 1. Highly represented Zur-bound regions (peaks) in the genome of *Streptomyces coelicolor*

peak	peak_ position	peak_ intensity	location	distance_ ORF	related gene_id	gene_ symbol	strand	gene description
peak_123	7378821	4.4097	internal	-487	SCO6643	-	+	hypothetical protein
peak_123	7378821	4.4097	intergenic	176	SCO6644	-	+	solute-binding lipoprotein
peak_023	2703951	4.3752	intergenic	108	SCO2504	glyS	-	glycyl-tRNA synthetase
peak_023	2703951	4.3752	intergenic	53	SCO2505	znuA	+	ABC-transporter lipoprotein
peak_124	3535835	4.2727	internal	-13	SCO3223	-	-	ABC transporter
peak_095	7508206	4.2402	intergenic	90	SCO6751	zitB	-	CDF type efflux protein
peak_095	7508206	4.2402	intergenic	388	SCO6752	-	+	integral membrane transferase
peak_040	6081704	4.1852	intergenic	121	SCO5579	-	-	transmembrane protein
peak_040	6081704	4.1852	intergenic	34	SCO5580	-	+	docking protein
peak_017	497296	4.1195	intergenic	79	SCO0476	znuC2	+	ABC transporter ATP-binding protein
peak_071	497296	4.1195	internal	-63	SCO0475	znuB2	-	ABC transporter protein
peak_050	3847121	4.0349	internal	-528	SCO3483	-	-	integral membrane transport protein
peak_157	3847121	4.0349	intergenic	431	SCO3482	-	-	sugar-permease
peak_024	4935364	3.9649	intergenic	257	SCO4511	-	-	hypothetical protein
peak_131	4935364	3.9649	internal	-321	SCO4512	-	-	hypothetical protein
peak_120	7867711	3.9486	internal	-587	SCO7077	-	-	hypothetical protein
peak_063	4151488	3.9347	intergenic	166	SCO3775	-	-	hypothetical protein
peak_103	4151488	3.9347	intergenic	360	SCO3777	-	+	peptidase
peak_155	4151488	3.9347	internal	-187	SCO3776	-	+	hypothetical protein
peak_016	5962697	3.9341	internal	-602	SCO5476	-	+	oligopeptide transport integral membrane protein
peak_040	5962697	3.9341	intergenic	477	SCO5477	-	+	putative oligopeptide-binding lipoprotein
peak_035	3935881	3.8549	intergenic	79	SCO3559	-	-	oxidoreductase
peak_035	3935881	3.8549	intergenic	24	SCO3560	-	+	ATP-binding protein
peak_156	665195	3.8451	internal	-578	SCO0624	-	-	hypothetical protein

peak_165	7898179	3.7833	internal	-85	SCO7108	-	-	hypothetical protein
peak_163	8001349	3.7229	internal	-988	SCO7197	-	-	amino acid ABC transporter permease
peak_009	3791129	3.6854	intergenic	26	SCO3428	rpmG	-	50S ribosomal protein L33
peak_011	3791129	3.6854	intergenic	197	SCO3427	rpmE2	-	50S ribosomal protein L31
peak_121	3791129	3.6854	intergenic	62	SCO3429	rpmB	+	50S ribosomal protein L28
peak_121	3791129	3.6854	intergenic	283	SCO3430	rpsN	+	30S ribosomal protein S14
peak_171	3791129	3.6854	intergenic	448	SCO3426	-	-	hypothetical protein
peak_019	6186618	3.6792	intergenic	173	SCO5682	-	-	hypothetical protein
peak_081	6186618	3.6792	internal	-30	SCO5683	-	+	two-component system sensor kinase
peak_061	6967646	3.6457	intergenic	172	SCO6307	-	-	cyclase
peak_061	6967646	3.6457	internal	-826	SCO6308	-	-	hydrolase
peak_071	457449	3.633	intergenic	35	SCO0436	rpmF	+	50S ribosomal protein L32
peak_085	5415283	3.5927	intergenic	41	SCO4977	-	-	TetR family transcriptional regulator
peak_050	3875941	3.554	internal	-1180	SCO3507	-	+	integral membrane efflux protein
peak_037	8224773	3.5521	internal	-4	SCO7409	-	-	binding-protein dependent transport protein
peak_049	5542240	3.5336	internal	-32	SCO5101	-	+	hypothetical protein
peak_098	5542240	3.5336	intergenic	235	SCO5102	-	+	mutT-like protein
peak_137	8506269	3.5171	intergenic	14	SCO7682	-	+	non-ribosomal peptide synthase
peak_161	8506269	3.5171	intergenic	147	SCO7681	-	-	AMP-binding ligase
peak_048	6237127	3.5143	intergenic	398	SCO5717	-	-	hypothetical protein
peak_048	6237127	3.5143	intergenic	81	SCO5718	-	+	hypothetical protein
peak_063	4135002	3.4911	intergenic	6	SCO3761	-	-	hypothetical protein
peak_027	1742190	3.4381	intergenic	23	SCO1627	cvnD9	-	ATP-GTP binding protein
peak_045	7520580	3.4336	intergenic	136	SCO6764	-	+	squalene-hopene cyclase
peak_062	7520580	3.4336	internal	-1117	SCO6763	-	+	polyprenyl synthatase
peak_104	1298023	3.4166	internal	-2308	SCO1226	-	-	hypothetical protein
peak_074	310453	3.406	internal	-75	SCO0313	-	+	transmembrane transport protein

peak_020	4304413	3.3977	internal	-155	SCO3908	rpsR	+	30S ribosomal protein S18
peak_164	4304413	3.3977	intergenic	100	SCO3909	rplI	+	50S ribosomal protein L9
peak_138	7495394	3.3126	internal	-1525	SCO6739	-	+	BCCT family transporter
peak_149	8498830	3.3078	internal	-418	SCO7675	-	+	hypothetical protein
peak_161	8498830	3.3078	intergenic	231	SCO7676	fdx3	+	Fdx3; ferredoxin
peak_039	4988778	3.2929	internal	-767	SCO4569	nuoH	+	NADH dehydrogenase subunit H
peak_145	7829456	3.277	internal	-2846	SCO7037	-	+	hypothetical protein
peak_028	3906872	3.2578	intergenic	397	SCO3539	-	+	transposase
peak_075	3906872	3.2578	intergenic	71	SCO3538	-	-	hypothetical protein
peak_105	5221376	3.2013	internal	-1530	SCO4798	-	-	peptidase
peak_003	7376994	3.1576	internal	-633	SCO6641	-	-	hypothetical protein
peak_032	4067752	3.1549	intergenic	358	SCO3684	-	-	hypothetical protein
peak_032	4067752	3.1549	intergenic	15	SCO3685	-	+	hypothetical protein
peak_104	1215993	3.1531	intergenic	66	SCO1154	-	-	hypothetical protein
peak_061	6986846	3.1424	intergenic	82	SCO6325	-	-	hypothetical protein
peak_158	6986846	3.1424	internal	-396	SCO6326	-	-	hypothetical protein
peak_022	8579982	3.1002	intergenic	74	SCO7747	-	+	hypothetical protein
peak_116	1448947	3.0809	internal	-666	SCO1372	-	-	hypothetical protein
peak_014	1052470	3.0623	intergenic	183	SCO0997	-	+	Fe uptake system integral membrane protein
peak_129	1052470	3.0623	internal	-857	SCO0996	-	+	lipoprotein
peak_057	7971873	3.0446	internal	-2161	SCO7173	-	+	transcriptional regulator
peak_002	5177153	3.037	internal	-48	SCO4764	-	-	dehydrogenase
peak_139	5138239	3.0202	internal	-584	SCO4722	secY	+	preprotein translocase subunit SecY
peak_091	6810573	2.9531	internal	-1774	SCO6198	-	+	hypothetical protein
peak_067	145377	2.9432	intergenic	11	SCO0154	-	-	hypothetical protein
peak_086	145377	2.9432	intergenic	122	SCO0155	-	+	transcriptional regulator
peak_040	6155367	2.9409	internal	-79	SCO5655	-	-	hypothetical protein

peak_105	5222983	2.9098	intergenic	77	SCO4798	-	-	lipase
peak_029	4990924	2.9095	intergenic	325	SCO4573	nuoL	+	NADH dehydrogenase subunit L
peak_115	4990924	2.9095	intergenic	11	SCO4572	nuoK	+	NADH dehydrogenase subunit K
peak_126	4990924	2.9095	internal	-843	SCO4571	nuoJ	+	NADH dehydrogenase subunit J
peak_106	7689045	2.9079	internal	-3	SCO6925	-	-	hypothetical protein
peak_055	6292782	2.9029	internal	-371	SCO5754	-	+	hypothetical protein
peak_059	6292782	2.9029	intergenic	284	SCO5755	-	+	hypothetical protein
peak_164	4457547	2.893	internal	-379	SCO4067	dnaZ	+	DNA polymerase III subunits gamma and tau
peak_006	6808501	2.8839	intergenic	298	SCO6198	-	+	hypothetical protein
peak_144	6808501	2.8839	intergenic	279	SCO6197	-	-	peptidase
peak_068	1179004	2.8827	internal	-710	SCO1120	-	+	hydrolase
peak_159	1179004	2.8827	intergenic	90	SCO1121	-	+	secreted protein
peak_083	5446147	2.8811	internal	-251	SCO5009	-	+	secretory protein
peak_021	5133444	2.875	intergenic	353	SCO4714	rplE	+	50S ribosomal protein L5
peak_052	5133444	2.875	internal	-341	SCO4712	rplN	+	50S ribosomal protein L14
peak_108	5133444	2.875	intergenic	30	SCO4713	rplX	+	50S ribosomal protein L24
peak_001	8640121	2.8743	internal	-84	SCO7821	-	+	hypothetical protein
peak_074	276945	2.8645	internal	-7	SCO0285	-	+	sodium/proton antiporter
peak_086	232478	2.8581	intergenic	30	SCO0244	-	+	hypothetical protein
peak_018	6474094	2.8342	intergenic	423	SCO5908	-	+	hypothetical protein
peak_042	6474094	2.8342	internal	-488	SCO5906	-	+	transcriptional regulator
peak_142	7954355	2.8106	internal	-306	SCO7159	-	+	hypothetical protein
peak_162	8018431	2.7866	internal	-590	SCO7214	-	-	hypothetical protein
peak_100	2929523	2.7858	intergenic	158	SCOt18	-	+	tRNA-Arg
peak_125	2929523	2.7858	intergenic	174	SCO2685	-	-	ATP-binding protein
peak_031	4205012	2.7785	intergenic	212	SCO3824	-	+	ABC transporter ATP-binding protein
peak_103	4205012	2.7785	internal	-69	SCO3823	-	-	quinone oxidoreductase

peak_167	1363264	2.774	intergenic	40	SCO1291	-	+	hypothetical protein
peak_012	1923218	2.7522	intergenic	449	SCO1792	-	-	3-methyladenine DNA glycosylase
peak_078	1923218	2.7522	internal	-302	SCO1793	-	+	Spo0M-homologous protein
peak_034	1650335	2.7509	intergenic	150	SCO1540	-	-	hypothetical protein
peak_136	1650335	2.7509	internal	-24	SCO1541	-	+	regulator
peak_092	3644512	2.7397	intergenic	349	SCO3294	-	-	transferase
peak_092	3644512	2.7397	internal	-423	SCO3295	-	-	oxidoreductase
peak_057	7982615	2.7358	internal	-216	SCO7182	-	+	branched-chain amino acid ABC transporter ATP-binding protein
peak_082	5449007	2.7293	intergenic	371	SCO5012	-	+	hypothetical protein
peak_084	5449007	2.7293	internal	-794	SCO5011	-	+	hypothetical protein
peak_073	1517280	2.7191	intergenic	104	SCO1421	-	+	hypothetical protein
peak_030	2689010	2.7103	internal	-894	SCO2495	-	+	hypothetical protein
peak_065	804719	2.7097	internal	-628	SCO0759	-	+	hypothetical protein
peak_065	804719	2.7097	intergenic	416	SCO0760	-	+	methyltransferase
peak_110	8436921	2.7059	intergenic	253	SCO7610	-	+	transcriptional regulator
peak_146	8436921	2.7059	internal	-53	SCO7609	-	-	esterase
peak_119	7839183	2.7003	intergenic	137	SCO7048	-	-	delta fatty acid desaturase
peak_119	7839183	2.7003	intergenic	24	SCO7049	-	+	glutaminase
peak_137	8552175	2.6866	internal	-245	SCO7717	-	-	hypothetical protein
peak_092	3648054	2.6667	internal	-494	SCO3299	-	-	hypothetical protein
peak_160	5829642	2.6435	intergenic	378	SCO5359	rpmE	+	50S ribosomal protein L31
peak_169	5829642	2.6435	internal	-895	SCO5358	-	+	hypothetical protein
peak_101	1143560	2.6291	internal	-5	SCO1082	-	-	electron transfer flavoprotein subunit beta
peak_041	7292023	2.6279	internal	-243	SCO6582	-	-	hypothetical protein
peak_087	2107213	2.6053	intergenic	14	SCO1966	-	-	excinuclease ABC subunit B
peak_151	6816835	2.5997	internal	-1338	SCO6201	-	+	glyoxylate carboligase
peak_122	6202094	2.5994	internal	-274	SCO5693	-	+	acyl CoA dehydrogenase

peak_116	1410198	2.5972	internal	-171	SCO1333	-	+	hypothetical protein
peak_167	1410198	2.5972	intergenic	476	SCO1332	-	-	hypothetical protein
peak_075	3876929	2.5837	intergenic	262	SCO3508	-	+	maturase-like protein
peak_008	3123795	2.5827	internal	-116	SCO2867	-	+	hypothetical protein
peak_113	3123795	2.5827	intergenic	119	SCO2868	-	+	hypothetical protein
peak_043	5739777	2.5806	internal	-1279	SCO5275	-	+	ATP/GTP binding protein
peak_114	860639	2.5768	internal	-676	SCO0812	-	+	sugar isomerase
peak_038	7591171	2.576	internal	-281	SCO6826	-	-	hypothetical protein
peak_047	25009	2.5541	intergenic	488	SCO0028	-	-	hypothetical protein
peak_047	25009	2.5541	intergenic	242	SCO0030	-	+	hypothetical protein
peak_010	6900407	2.5427	internal	-1237	SCO6272	-	+	FAD-binding protein
peak_007	5561003	2.536	internal	-589	SCO5114	bldKC	+	ABC transporter integral membrane protein BldKC
peak_007	5561003	2.536	intergenic	399	SCO5115	bldKD	+	ABC transporter intracellular ATPase subunit BldKD
peak_008	3096784	2.5321	internal	-1278	SCO2836	-	+	glycosyl transferase
peak_135	64284	2.4666	intergenic	191	SCO0072	-	-	hypothetical protein
peak_135	64284	2.4666	intergenic	321	SCO0073	-	+	hypothetical protein
peak_109	4670524	2.4534	internal	-392	SCO4258	-	+	hydrolytic protein
peak_064	4667461	2.4531	internal	-385	SCO4256	-	+	hydrolytic protein
peak_109	4669011	2.4476	internal	-420	SCO4257	-	+	hydrolytic protein
peak_168	4621170	2.4314	internal	-79	SCO4213	-	+	hypothetical protein
peak_093	2924383	2.4197	intergenic	91	SCO2682	-	-	hypothetical protein
peak_051	5321689	2.4092	internal	-372	SCO4887	-	+	sugar ABC transporter integral membrane protein
peak_015	104769	2.3993	internal	-1141	SCO0124	-	+	hypothetical protein
peak_067	104769	2.3993	intergenic	288	SCO0125	-	+	oxidoreductase
peak_010	6873779	2.3861	intergenic	70	SCO6251	-	+	reductase
peak_029	5009006	2.3857	internal	-519	SCO4586	-	+	ABC transporter
peak_026	2553566	2.379	internal	-1310	SCO2383	-	+	hypothetical protein

peak_022	8563938	2.3757	internal	-313	SCO7729	-	+	hypothetical protein
peak_099	8315573	2.3643	internal	-142	SCO7502	-	-	LacI family transcriptional regulator
peak_143	8315573	2.3643	intergenic	333	SCO7503	-	+	extracellular solute-binding protein
peak_152	652485	2.3617	internal	-883	SCO0613	arcA	+	arginine deiminase
peak_070	2406200	2.3358	internal	-258	SCO2235	-	+	hypothetical protein
peak_070	2406200	2.3358	intergenic	2	SCO2236	-	+	hypothetical protein
peak_096	2406200	2.3358	intergenic	319	SCO2237	-	+	lyase
peak_147	2406200	2.3358	intergenic	432	SCO2234	glnE	-	bifunctional glutamine-synthetase adenylyltransferase/deadenyltransferase
peak_065	791520	2.3353	internal	-15	SCO0749	-	-	dehydratase
peak_148	4608672	2.3326	internal	-113	SCO4200	-	+	hypothetical protein
peak_018	6603031	2.3242	internal	-521	SCO6020	-	-	transcriptional regulator
peak_094	2768537	2.3235	internal	-7	SCO2565	-	-	hypothetical protein
peak_154	2768537	2.3235	intergenic	259	SCO2564	-	-	DNA-binding protein
peak_020	4274048	2.3204	internal	-16	SCO3883	-	+	inner membrane protein translocase component YidC
peak_088	3204771	2.3182	intergenic	124	SCO2948	-	-	hypothetical protein
peak_088	3204771	2.3182	intergenic	101	SCO2949	murA	+	UDP-N-acetylglucosamine 1-carboxyvinyltransferase
peak_124	3467692	2.3135	internal	-1767	SCO3166	-	-	membrane transport protein
peak_112	75766	2.3078	internal	-110	SCO0088	-	-	beta-lactamase
peak_112	75766	2.3078	intergenic	90	SCO0089	-	+	transcriptional regulator
peak_069	5075936	2.3075	internal	-663	SCO4651	-	+	lipoprotein
peak_072	2479602	2.2996	internal	-39	SCO2309	-	-	transmembrane transport protein
peak_072	2479602	2.2996	intergenic	315	SCO2310	-	+	integral membrane efflux protein
peak_065	703925	2.2923	internal	-175	SCO0665	-	+	hypothetical protein
peak_065	703925	2.2923	intergenic	189	SCO0666	catB	+	catalase
peak_055	6253563	2.2855	internal	-3809	SCO5734	-	-	ATP/GTP binding protein membrane protein
peak_077	6253563	2.2855	intergenic	228	SCO5733	-	-	hypothetical protein
peak_013	7718901	2.278	internal	-540	SCO6953	-	-	hypothetical protein

peak_052	5123915	2.2683	internal	-60	SCO4699	-	+	Rhs protein
peak_015	79753	2.263	intergenic	51	SCO0095	-	-	hypothetical protein
peak_058	7877768	2.2626	internal	-1813	SCO7090	-	-	hypothetical protein
peak_074	269393	2.2561	intergenic	390	SCO0278	-	+	MutT domain-containing protein
peak_150	269393	2.2561	internal	-295	SCO0277	-	-	hypothetical protein
peak_025	3602351	2.2515	intergenic	273	SCO3248	fabF3	-	3-oxoacyl-ACP synthase
peak_025	3602351	2.2515	intergenic	31	SCO3249	-	-	acyl carrier protein
peak_053	3602351	2.2515	intergenic	258	SCOt27	-	+	tRNA-Arg
peak_172	8045120	2.2431	internal	-369	SCO7236	qcrB3	-	ubiquinol-cytochrome C reductase cytochrome subunit B
peak_132	2418880	2.238	internal	-127	SCO2251	-	+	hypothetical protein
peak_060	58507	2.232	internal	-2282	SCO0069	-	+	hypothetical protein
peak_117	6783780	2.2144	internal	-31	SCO6178	-	-	deacetylase
peak_117	6783780	2.2144	intergenic	203	SCO6179	-	+	nucleotide-sugar dehydratase
peak_036	7550869	2.208	internal	-1408	SCO6790	-	+	long chain fatty acid CoA ligase
peak_002	5212045	2.1943	intergenic	123	SCO4790	-	-	hypothetical protein
peak_128	5212045	2.1943	intergenic	68	SCO4791	-	+	two-component system sensor kinase
peak_102	3603229	2.1855	internal	-678	SCO3250	-	-	integrase
peak_130	2672012	2.1778	intergenic	71	SCO2481	-	-	hypothetical protein
peak_170	2672012	2.1778	intergenic	37	SCO2482	-	+	dehydrogenase
peak_078	2000049	2.174	internal	-939	SCO1865	-	+	diaminobutyrate--2-oxoglutarate aminotransferase
peak_134	2000049	2.174	intergenic	451	SCO1866	ectC	+	L-ectoine synthase
peak_166	2074770	2.1729	internal	-433	SCO1941	-	-	hypothetical protein
peak_153	3842242	2.1423	internal	-1140	SCO3479	-	-	beta-galatosidase
peak_127	5566590	2.1402	internal	-131	SCO5119	-	+	peptide transport system integral membrane protein
peak_089	5698082	2.1291	intergenic	6	SCO5236	nagB	-	glucosamine-6-phosphate deaminase
peak_148	4543587	2.1281	internal	-724	SCO4129	-	-	hypothetical protein
peak_111	365629	2.1221	intergenic	78	SCO0360	-	-	LacI family DNA-binding transcriptional repressor

peak_111	365629	2.1221	internal	-1166	SCO0361	-	-	hypothetical protein
peak_141	3335815	2.1216	internal	-946	SCO3045	-	+	hypothetical protein
peak_088	3183027	2.1216	internal	-615	SCO2931	-	+	ABC transporter ATP-binding protein
peak_054	7736044	2.1178	intergenic	63	SCO6969	-	+	hypothetical protein
peak_056	7736044	2.1178	internal	-1535	SCO6968	-	+	long-chain-fatty-acid-CoA ligase
peak_005	6467391	2.1085	internal	-1764	SCO5900	-	+	hypothetical protein
peak_005	6467391	2.1085	intergenic	364	SCO5901	-	+	RNA methyltransferase
peak_042	6470959	2.0872	internal	-2493	SCO5905	-	-	export protein
peak_090	6470959	2.0872	intergenic	85	SCO5904	-	-	hypothetical protein
peak_079	3424042	2.0572	internal	-53	SCO3123	prsA2	+	ribose-phosphate pyrophosphokinase
peak_140	8475943	2.0571	intergenic	62	SCO7648	-	-	two-component system response regulator
peak_107	7705183	2.0461	internal	-6	SCO6940	cvnD8	-	ATP/GTP binding protein
peak_118	3402026	2.0455	intergenic	224	SCO3104	-	-	hypothetical protein
peak_118	3402026	2.0455	internal	-127	SCO3105	-	+	hypothetical protein
peak_076	8378969	2.0397	internal	-268	SCO7556	-	+	sugar transport permease
peak_044	8357292	2.0336	internal	-1753	SCO7536	-	+	hypothetical protein
peak_001	8652981	2.0226	intergenic	73	SCO7835	-	+	hypothetical protein
peak_166	2062048	2.0006	internal	-143	SCO1930	-	+	integral membrane transport protein
peak_004	5353579	1.9989	internal	-992	SCO4919	-	+	flavoprotein disulfide reductase
peak_013	7705714	1.9961	internal	-450	SCO6942	cvnB8	-	hypothetical protein
peak_080	7705714	1.9961	intergenic	62	SCO6941	cvnC8	-	hypothetical protein
peak_136	1721542	1.9915	internal	-919	SCO1610	-	-	hypothetical protein
peak_130	2636293	1.9865	internal	-3498	SCO2450	-	+	Ser/Thr protein kinase (regulator)
peak_046	4270021	1.9825	intergenic	178	SCO3878	dnaN	-	DNA polymerase III subunit beta
peak_145	7809078	1.9732	intergenic	146	SCO7023	-	+	hypothetical protein
peak_160	5863144	1.9716	internal	-412	SCO5394	-	-	ABC transporter
peak_066	7650506	1.9684	internal	-825	SCO6881	-	-	ATP-binding protein

peak_120	7841298	1.9639	internal	-105	SCO7050	-	-	D-alanyl-D-alanine carboxypeptidase
peak_033	8318793	1.945	intergenic	381	SCO7506	-	+	hydrolase
peak_143	8318793	1.945	internal	-584	SCO7505	-	+	integral membrane binding-protein-dependent transport protein
peak_036	7533909	1.9292	intergenic	193	SCO6776	-	+	hypothetical protein
peak_045	7533909	1.9292	intergenic	40	SCO6775	-	-	transcriptional regulator
peak_114	1047957	1.9265	internal	-846	SCO0992	-	+	cysteine synthase
peak_129	1047957	1.9265	intergenic	173	SCO0993	-	+	hypothetical protein
peak_133	14477	1.8949	intergenic	454	SCO0011	-	-	hypothetical protein
peak_133	14477	1.8949	intergenic	23	SCO0012	-	-	hypothetical protein
peak_004	5354818	1.8837	internal	-589	SCO4920	-	+	DeoR family transcriptional regulator
peak_097	3165087	1.8588	internal	-187	SCO2914	-	-	amino acid permease

From 3 sites **TGaaAatgatTttCA** **(Shin *et al.,* 2007)**

From 6 sites **ATGAaNNTCaTTTTCA** **(Kallifidas *et al.,* 2010)**

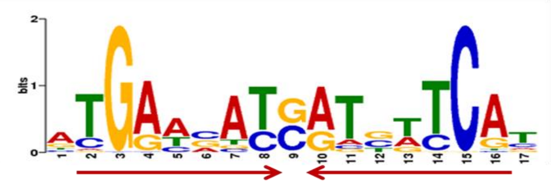

tGaNNatSatNNtCa **(This study)**

zitB TGACAACGGTGTTCA

Actinobacterial *znuA* taaTGaNAANNNTTNtCANta **(Schroder et al., 2010)**

Supplementary Figure 1. Comparison of Zur-binding consensus sequences. The Zurbox consensus sequences determined from three Zur-bound sites (Shin et al. 2007)[1], from 6 bound sites (Kallifidas et al., 2010)[2], and this study (172 sites, E-value of 3.9e-233). The *zitB* Zurbox sequence was shown below, along with computational Zur-box consensus obtained from *znuA* genes of 17 actinobacterial genomes (Schroder et al., 2010)[3].

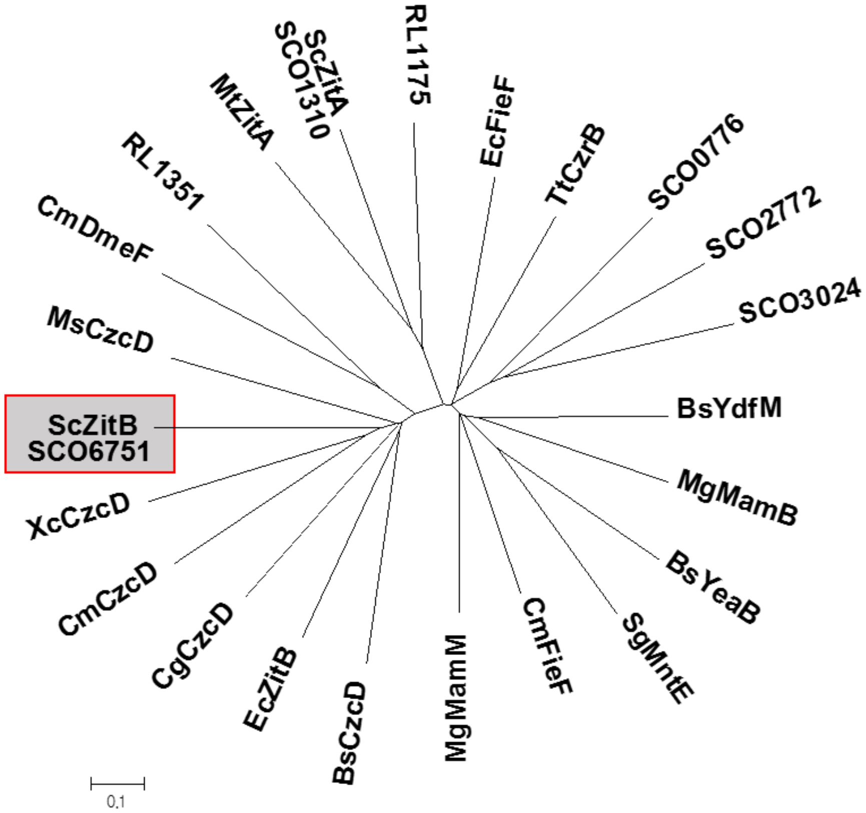

Supplementary Figure 2. Phylogenetic relationship of SCO6751 with other bacterial CDF-type transporters. We compared the sequence of SCO6751 with those of several CDF-type efflux pumps of reported functions. The selected ones include $Zn^{2+}/Cd^{2+}/Fe^{2+}$ exporter FieF of *E. coli*, $Zn^{2+}/Cd^{2+}/Ni^{2+}/Cu^{2+}$ exporter ZitB of *E. coli*, and others such as ZitA of *Mycobacterium tuberculosis*, CzrB of *Thermus thermophilus*, MamM of *Magnetospirillum. gryphiswaldense* MSR-1, CzcDs of *B. subtilis* and *M. tuberculosis*[4, 5, 6, 7, 8]. These sequences were aligned with Clustal W algorithm and visualized with MEGA7 software. Abbreviations and sequence sources are as follows. From *B. subtilis,* BsCzcD[9] (NP_390542), BsYdfM[10] (NP_388428), BsYeaB[11] (NP_388513) ; from *Corynebacterium glutamicum*, CgCzcD[12] (NP_600503); from *Cupriavidus metallidurans*, CmCzcD[13] (CAA67085), CmDmeF[14] (ABF07084), CmFieF[14] (ABF10278); from *E. coli*, EcFieF (NP_418350), EcZitB(NP_415273); from *M. gryphiswaldense*, MgMamB[15] (CDK99583),

MgMamM[15] (CAJ30120); from *M. smegmatis*, MsCzcD[16] (YP_885161); from *M. tuberculosis H37Rv*, MtZitA[17] (NP_216541); from *Rhizobium leguminosarum*, RL1175[18] (CAK06672), RL1351[18] (CAK06848); from *S. coelicolor A3(2)*, SCO0776 (NP_625078), SCO2772(NP_627002), SCO3024 (NP_627246), SCO1310 (NP_625596), SCO6751 (ScZitB; NP_630824); from *T. thermophiles*, TtCzrB[19] (CAC83722); from *Xanthomonas campestris*, XcCzcD[20] (WP_011036459).

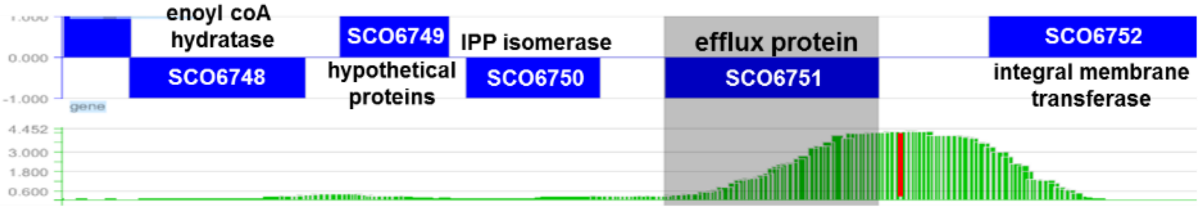

Supplementary Figure 3. The location of Zur-box on the *zitB* gene relative to the transcription start site (TSS). The ChIP-chip data for Zur binding near the SCO6751 gene. The position of Zur-box motif was shown in red in the middle of a broad peak of Zur-bound DNAs (green).

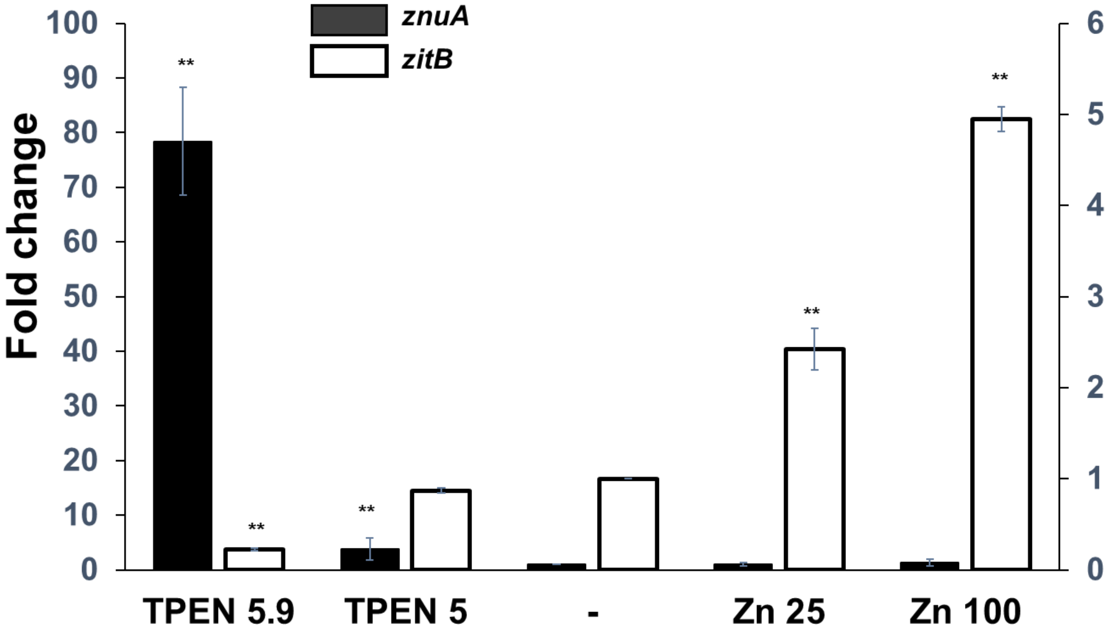

Supplementary Figure 4. Quantification of the *znuA* and *zitB* RNAs by qRT-PCR. The same RNA samples that were used in S1 mapping were evaluated by qRT-PCR. The forward (5'-GAACGTACGACGACGCCGCA-3') and reverse (5'-CCGAGCTGTCGCTGGAGCAG-3') primers were used to for znuA. The forward (5'-GGTACACACGAGGAGGCGGC-3') and reverse (5'-GTGATCGACAGCGCCACGCG-3') primers were used to for *zitB*. The qRT-PCR values were normalized to the constant reference gene (SCO0710) [21], and were presented as relative fold changes, by taking the untreated sample values as 1.0. Three independent RNA samples were obtained for qPCR analysis, using the Stratagene MX3000P QPCR system (Agilent Technologies). Average values with standard deviations (error bars) were presented. Asterisks (**) indicate measurements with P-values ≤0.001 by Student's t-test.

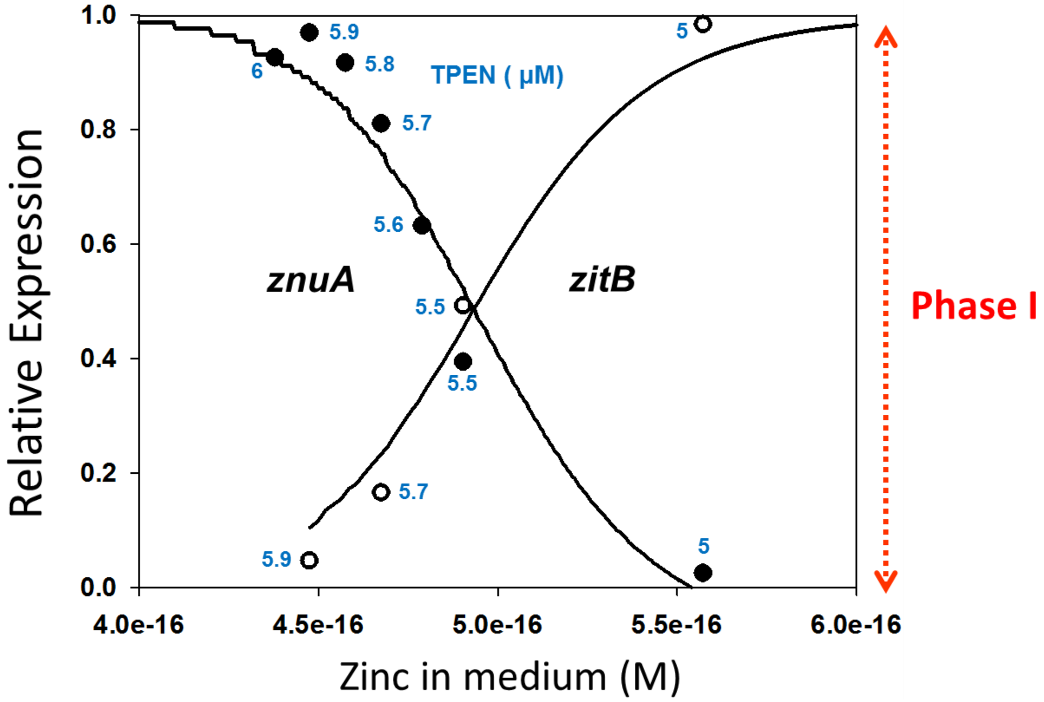

Supplementary Figure 5. The zinc-dependent expression of the *zitB* and znuA gene at sub-femtomolar range of zinc concentrations. For both genes, the maximal level of expression was taken as 1.0 for cells treated with TPEN (from 5 to 6 μM) in YEME medium. For the *zitB* gene, the maximal level of expression at phase I activation range occurred when TPEN was treated at 5 μM.

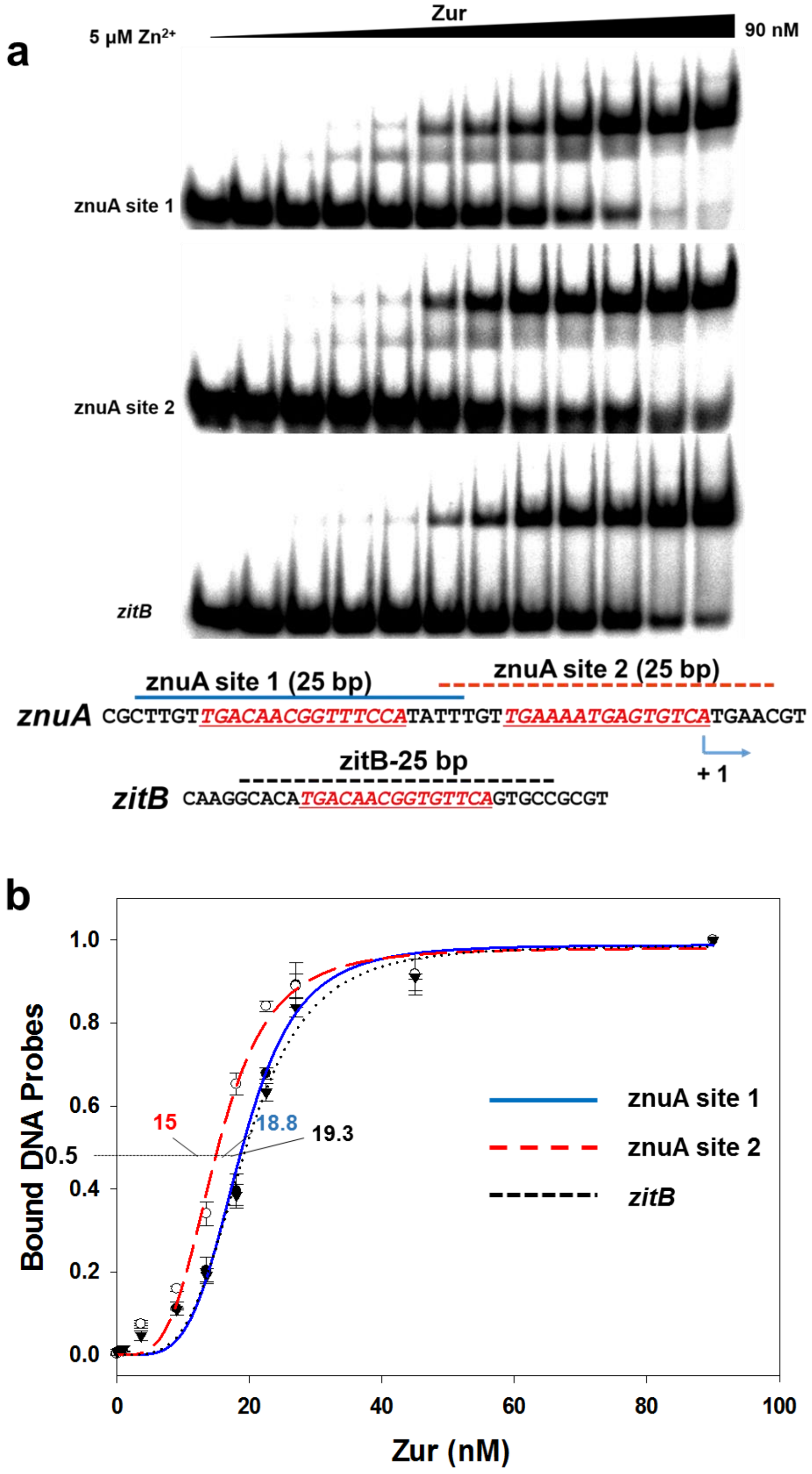
a
5 μM Zn2+
Zur
90 nM
znuA site 1
znuA site 2
zitB
znuA site 1 (25 bp)
znuA site 2 (25 bp)
znuA CGCTTGTTGACAACGGTTTCCATATTTGTTGAAAATGAGTGTCATGAACGT
+ 1
zitB-25 bp
zitB CAAGGCACATGACAACGGTGTTCAGTGCCGCGT
b
Bound DNA Probes
Zur (nM)
15
18.8
19.3
0.5
znuA site 1
znuA site 2
zitB

Supplementary Figure 6. Binding of Zur to 25 bp DNA probes containing the core Zur-box motifs (in red italic) of the *znuA* (site 1 and site 2) or *zitB* promoters. (**a**) EMSA between Zur and 25 bp DNA probes. Increasing amounts of purified Zur protein (0.9, 1.8, 2.7, 3.6, 9, 13.5, 18, 22.5, 27, 45 and 90 nM) were incubated in the presence of 5 μM $ZnSO_4$ with γ-^{32}P labeled synthetic DNA probes for *zitB*, *znuA* site1 and *znuA* site2 as indicated at the bottom. (**b**) The binding curves (Hill plots) were generated from EMSA data for the *znuA* site 1 (filled circle, blue line), *znuA* site 2 (open circle, red dotted line), and *zitB* (filled triangle, black dotted line) probes. The Kd values were estimated to be 18.8, 15, and 19.3 nM for *znuA* site 1, *znuA* site 2, and *zitB*, respectively. Average values from three independent experiments were presented with standard deviations.

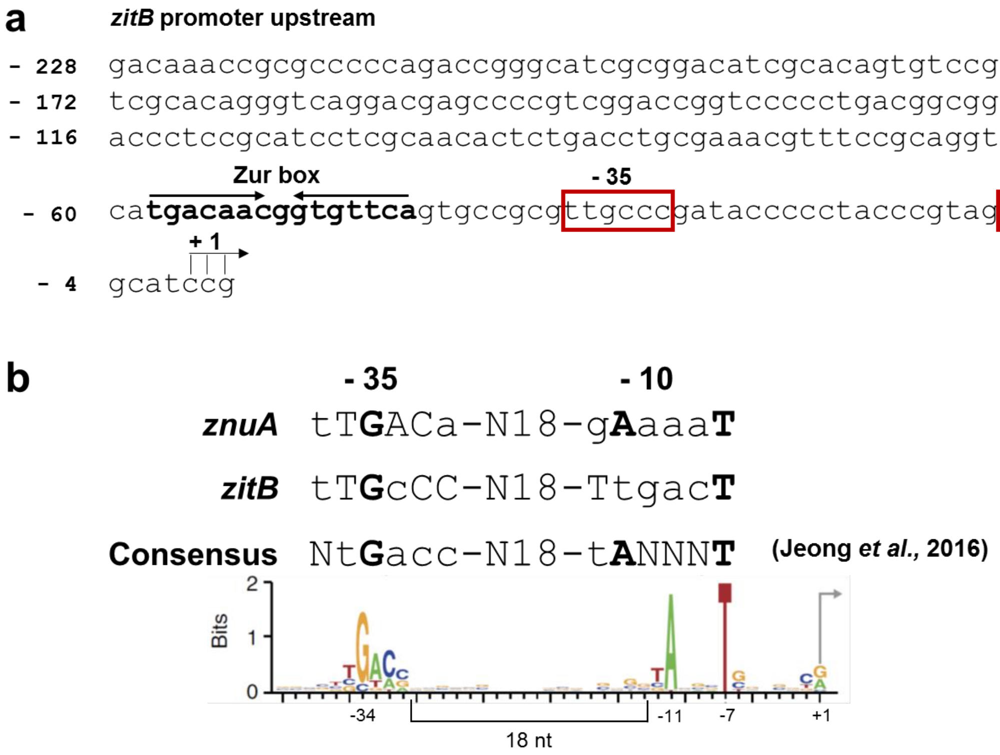

Supplementary Figure 7. **Sequence features of the *zitB* promoter region.** (**a**) Promoter upstream sequences of the *zitB* gene. (**b**) Comparison of the -35 and -10 elements of the *zitB* promoter with those of *znuA* and the prominent promoters, whose transcription start sites (TSS) have been determined in the genome of *S. coelicolor* [22]. The bold capitals denote most conserved nucleotides. The nucleotide positions in the prominent promoter consensus logo were numbered based on the most frequently observed distances from the TSS[22].

.

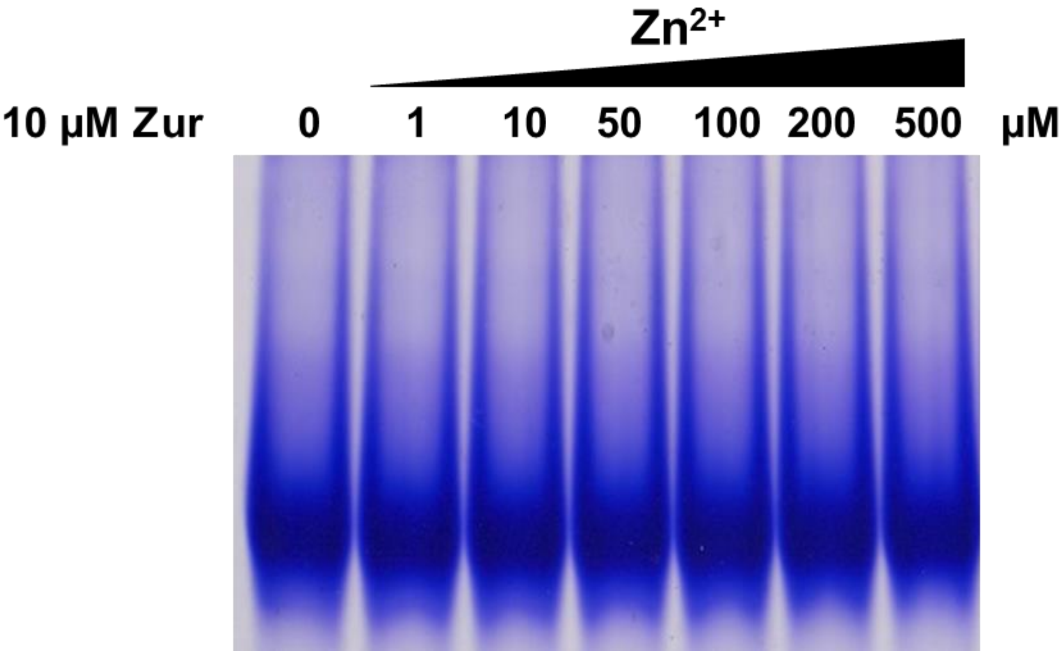

Supplementary Figure 8. **Constant electrophoretic mobility of Zur on native PAGE over a wide range of zinc concentration changes.** Increasing amounts of zinc (0 to 0.5 mM) were added to 10 μM Zur in the binding buffer, whose electrophoretic mobility change was examined on 5 % native PAGE.

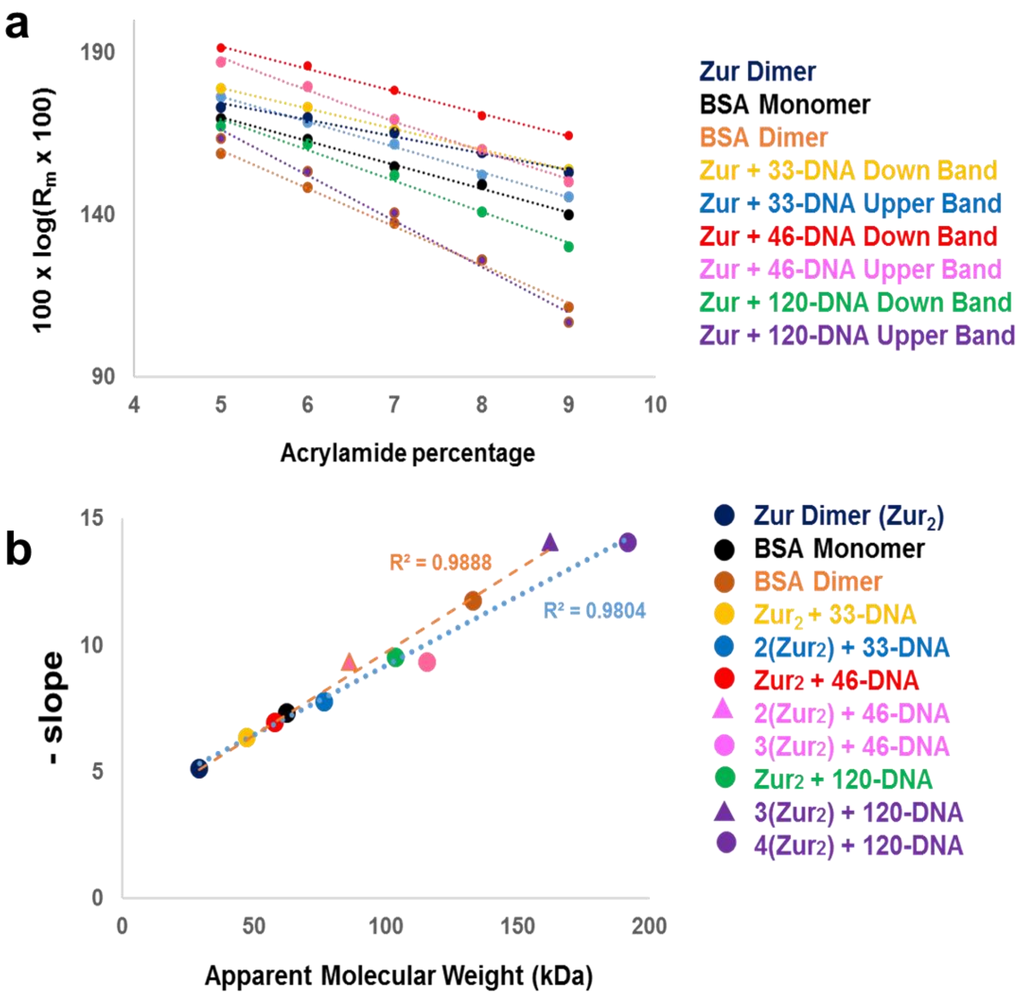

Supplementary Figure 9. Determination of the stoichiometry of Zur-DNA complexes by native PAGE mobility. (a) Relative mobility of Zur-DNA complexes on DNA probes of 33, 46, or 120 bp probes on native PAGE on 5 to 9% polyacrylamide gels. Logarithms of relative mobility values (compared with bromophenol blue) of both upper and down complexes as well as standard proteins (BSA monomer, BSA dimer, Zur dimer) were plotted against acylamide percentages as described by Baichoo and Helmann (2002)[23]. Data points for each complex and standard protein were presented with color-coded legends. (b) Estimation of molecular mass and the stoichiometry of Zur-DNA complex. The negative slope of the mobility change for each standard protein was re-plotted

against their molecular weights to obtain a standard curve. The apparent molecular weight of each complex was estimated from the standard curve using the least squares regression. The best fitting line (R^2 of 0.9888; brown dotted line) was obtained by assuming the following stoichiometry of Zur for upper and down complex bands, respectively: On 33 bp DNA, tetrameric (47.2 kDa, blue) and dimeric (76.6 kDa, orange) Zur binding; on 46 bp DNA, tetrameric (86.3 kDa, pink triangle) and dimeric (57.9 kDa, red circle) Zur binding; On 120 bp probe, hexameric (162.4 kDa, purple triangle) and dimeric (103.6 kDa, green circle) binding. Another linear regression of data plots gave reasonably well-fitting curve (R^2 of 0.9804; blue dotted line), when the upper bands on longer probes (46 bp and 120 bp) were regarded as hexameric (115.7 kDa with 46 bp DNA; pink circle) and octameric (191.8 kDa with 120 bp DNA; purple circle) Zur-bound complexes, respectively.

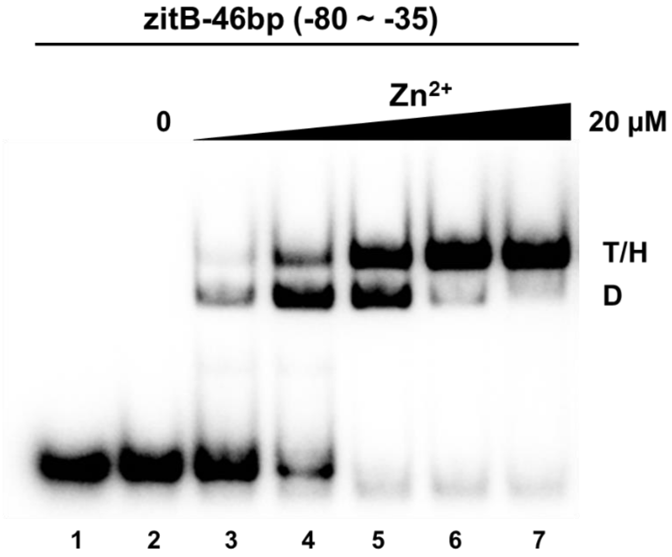

Supplementary Figure 10. Binding of Zur to 46 bp DNA (from -80 to -35). EMSA assay was performed for the binding reactions containing Increasing amounts of zinc (0, 0.5, 1, 5, 10, 20 μM) and 90 nM Zur. The upper and down complexes were labeled as T/H for tetramer or hexamer, and D for dimer, as estimated from the PAGE analysis on different acrylamide percentages (Supplementary Fig. 9).

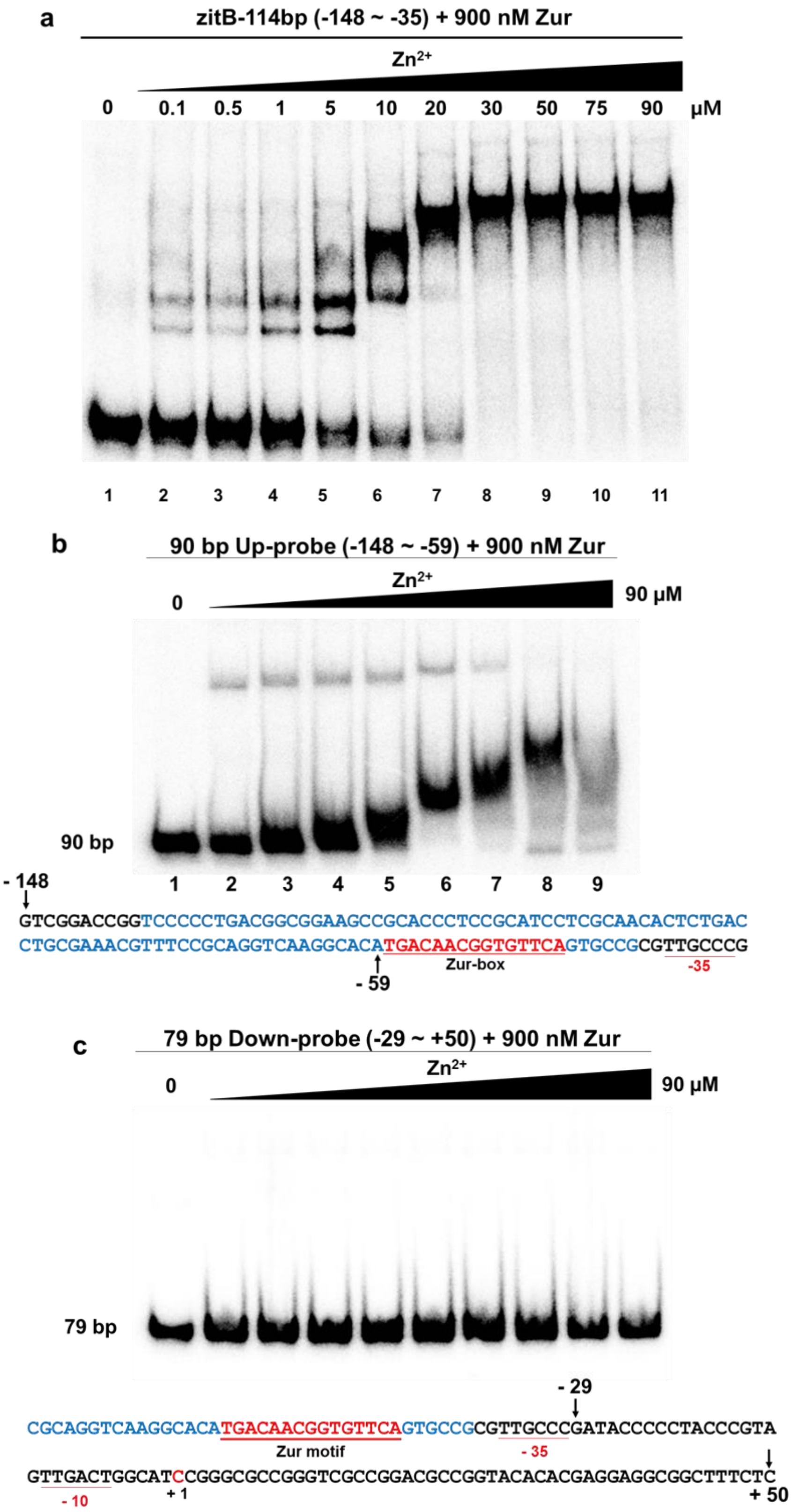
a
zitB-114bp (-148 ~ -35) + 900 nM Zur
Zn2+
0 0.1 0.5 1 5 10 20 30 50 75 90 μM
1 2 3 4 5 6 7 8 9 10 11
b
90 bp Up-probe (-148 ~ -59) + 900 nM Zur
Zn2+
0
90 μM
90 bp
1 2 3 4 5 6 7 8 9
- 148
GTCGGACCGGTCCCCCTGACGGCGGAAGCCGCACCCTCCGCATCCTCGCAACACTCTGAC
CTGCGAAACGTTTCCGCAGGTCAAGGCACATGACAACGGTGTTCAGTGCCGCGTTGCCCG
Zur-box
-35
- 59
c
79 bp Down-probe (-29 ~ +50) + 900 nM Zur
Zn2+
0
90 μM
79 bp
- 29
CGCAGGTCAAGGCACATGACAACGGTGTTCAGTGCCGCGTTGCCCGATACCCCCTACCCGTA
Zur motif
- 35
GTTGACTGGCATCCGGGCGCCGGGTCGCCGGACGCCGGTACACACGAGGAGGCGGCTTTCTC
- 10
+ 1
+ 50

Supplementary Figure 11. Zinc-dependent binding of Zur to 114 bp *zitB* probe at high concentration of Zur (900 nM). (**a**) EMSA of Zur binding to 114 bp probe (from -148 to -35 nt from TSS) at 900 nM Zur with increasing concentrations of zinc (0.1, 0.5, 1, 5, 10, 20, 30, 50, 75, 90 µM) in the binding reaction. Formation of super-shifted bands appear at >10 µM zinc. (**b**) EMSA of Zur binding (900 nM) to upstream DNA probe (-148 to -59) devoid of Zur-box motif. The same binding conditions as in (a) were used. (**c**) EMSA of Zur binding (900 nM) to downstream DNA probe (-29 to +50) devoid of Zur-box motif. The same binding conditions as in (a) were used.

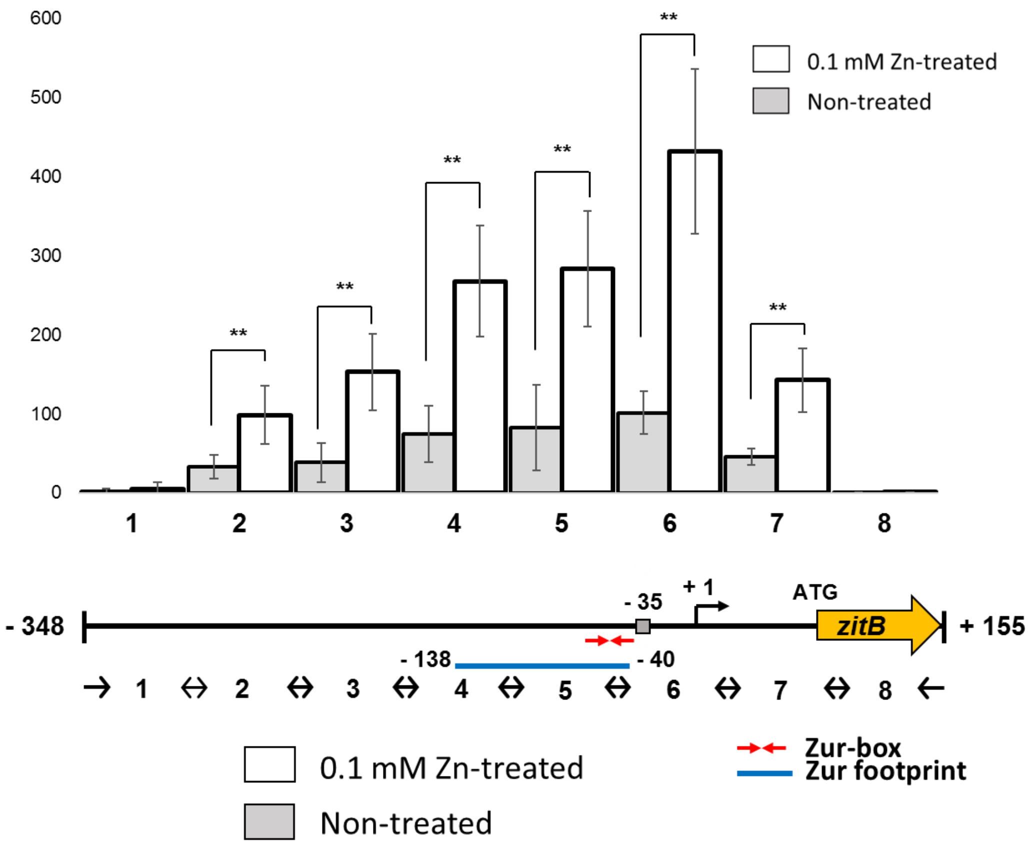

Supplementary Figure 12. ChIP analysis of zinc-dependent binding of Zur around the *zitB* promoter region *in vivo*. Quantitative PCR (qPCR) was performed for the chromatin samples immunoprecipitated with anti-Zur antibody. Wild type cells were either non-treated or treated with 0.1 mM $ZnSO_4$. Overlapping primers were used to for regions 1 (88 bp, -348 ~ -260), 2 (72 bp, -280 ~ -208), 3 (71 bp, -228 ~ -157), 4 (87 bp, -177 ~ -90), 5 (75 bp, -110 ~ -35), 6 (94 bp, -55 ~ +39), 7 (65 bp, +18 ~ +83), and 8 (93 bp, +62 ~ +155). Relative enrichments were presented by taking the value from *Δzur* sample as 1.0. Three independent ChIP samples were obtained for qPCR analysis, using the Stratagene MX3000P QPCR system (Agilent Technologies). Average values with standard deviations were presented. Asterisks (**) indicate measurements with P-values ≤ 0.001 by Student's t-test.

Fig. 1a

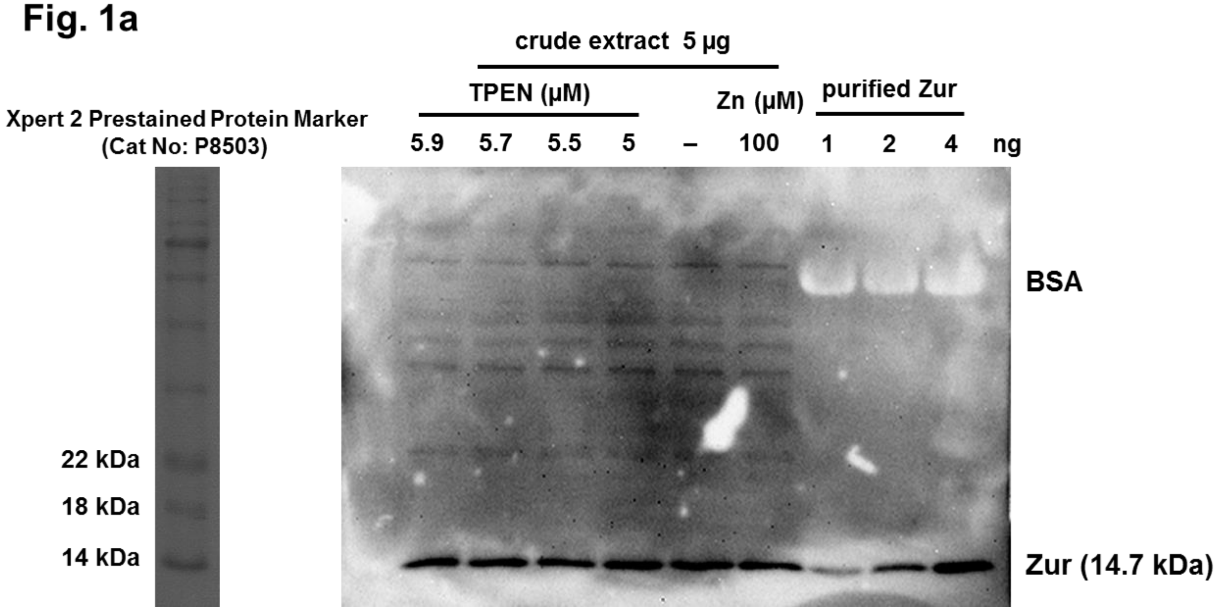

Representative full-sized S1 mapping gel for *zitB* and *znuA* transcripts

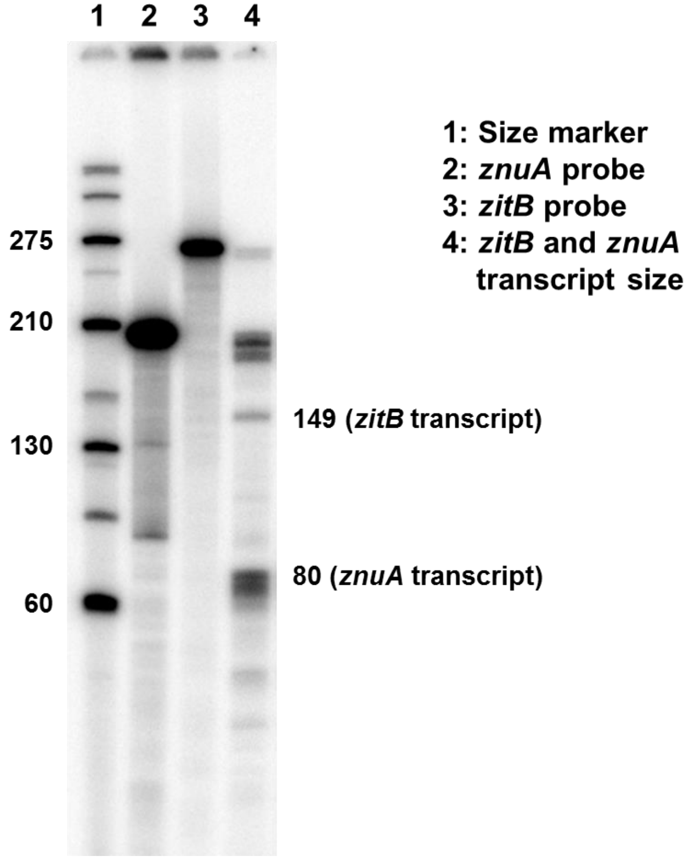

Fig. 1d

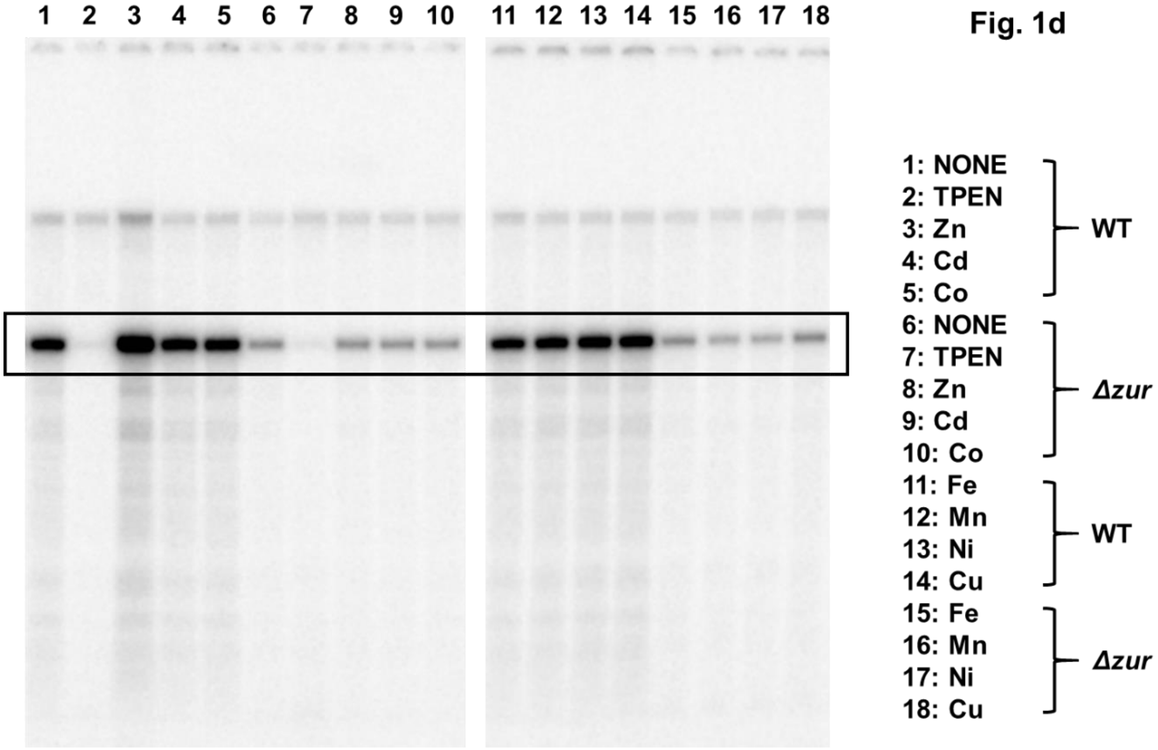
1 2 3 4 5 6 7 8 9 10 11 12 13 14 15 16 17 18
1: NONE
2: TPEN
3: Zn
4: Cd
5: Co
WT
6: NONE
7: TPEN
8: Zn
9: Cd
10: Co
Δzur
11: Fe
12: Mn
13: Ni
14: Cu
WT
15: Fe
16: Mn
17: Ni
18: Cu
Δzur

Fig. 2b

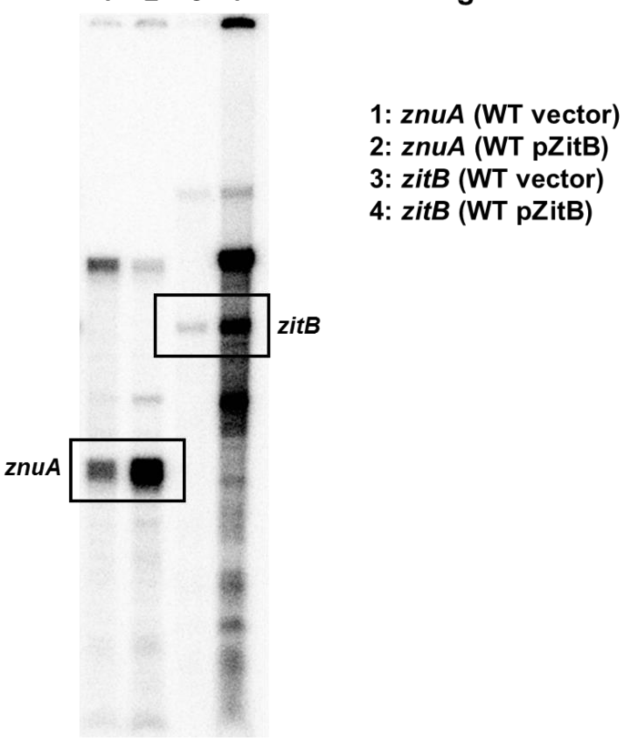
1 2 3 4
1: znuA (WT vector)
2: znuA (WT pZitB)
3: zitB (WT vector)
4: zitB (WT pZitB)
zitB
znuA

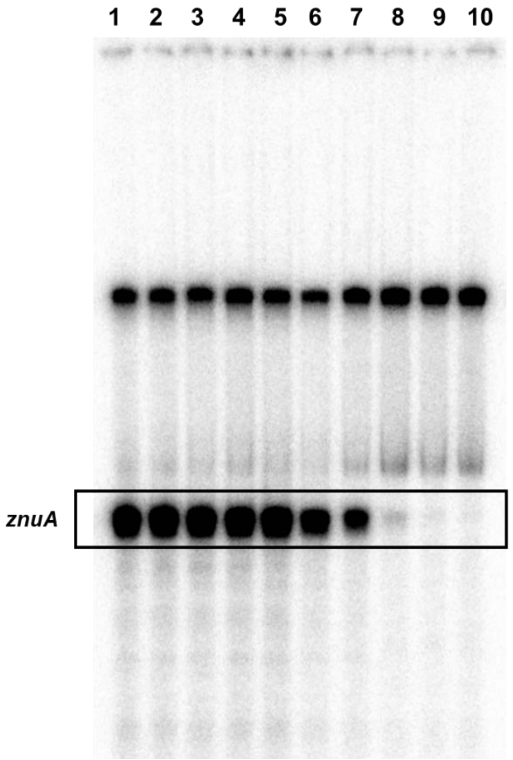

Fig. 3a

1: TPEN 6.5
2: TPEN 6
3: TPEN 5.9
4: TPEN 5.8
5: TPEN 5.7
6: TPEN 5.6
7: TPEN 5.5
8: TPEN 5
9: NONE
10: Zn 100

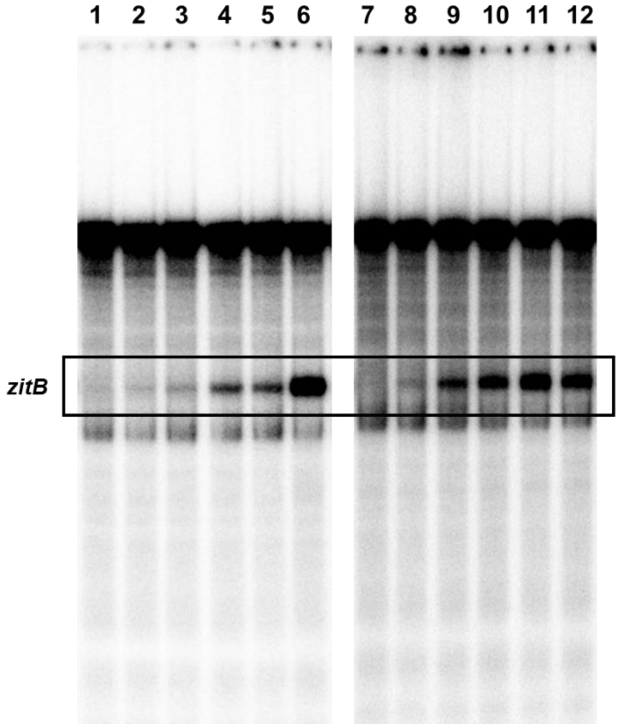

Fig. 3a

1: TPEN 5.9
2: TPEN 5.7
3: TPEN 5.5
4: TPEN 5
5: NONE
6: Zn 100
7: TPEN 6.5
8: NONE
9: Zn 25
10: Zn 50
11: Zn 100
12: Zn 150

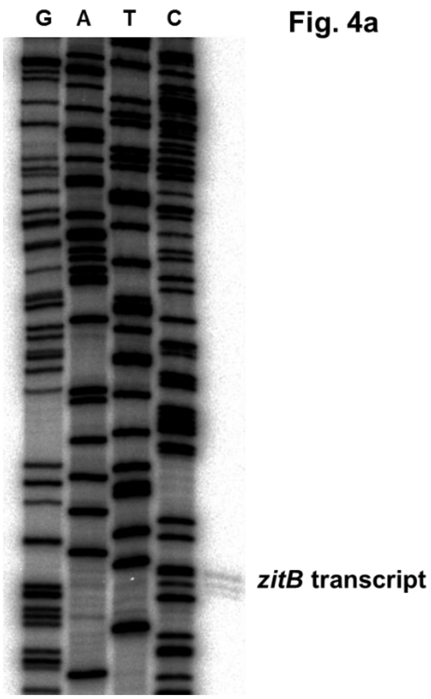

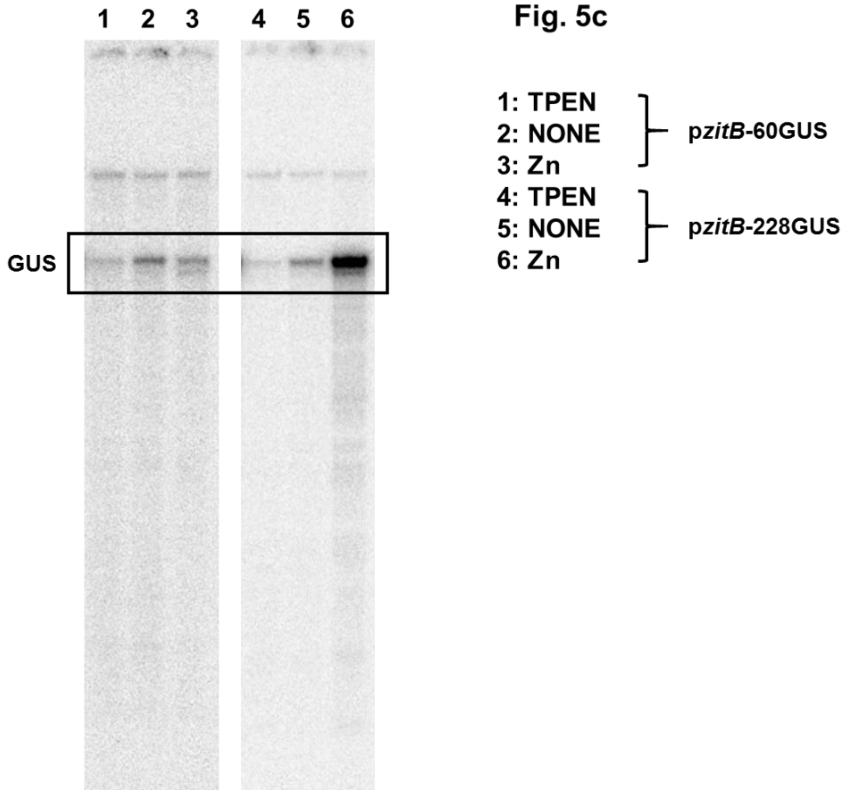

Supplementary Figure 13. Full-sized photos of Western blots and S1 mapping gels presented in the figures.

Supplementary References

1. Shin JH, Oh SY, Kim SJ, Roe JH. The zinc-responsive regulator Zur controls a zinc uptake system and some ribosomal proteins in *Streptomyces coelicolor* A3(2). *Journal of bacteriology* **189**, 4070-4077 (2007).

2. Kallifidas D, Pascoe B, Owen GA, Strain-Damerell CM, Hong HJ, Paget MS. The zinc-responsive regulator Zur controls expression of the coelibactin gene cluster in *Streptomyces coelicolor*. *Journal of bacteriology* **192**, 608-611 (2010).

3. Schroder J, Jochmann N, Rodionov DA, Tauch A. The Zur regulon of *Corynebacterium glutamicum ATCC 13032*. *BMC Genomics* **11**, 12 (2010).

4. Chao Y, Fu D. Thermodynamic studies of the mechanism of metal binding to the *Escherichia coli* zinc transporter YiiP. *J Biol Chem* **279**, 17173-17180 (2004).

5. Paulsen IT, Saier MH, Jr. A novel family of ubiquitous heavy metal ion transport proteins. *J Membr Biol* **156**, 99-103 (1997).

6. Rahman M*, et al.* Probing metal ion substrate-binding to the *E. coli* ZitB exporter in native membranes by solid state NMR. *Mol Membr Biol* **25**, 683-690 (2008).

7. Wei Y, Fu D. Selective metal binding to a membrane-embedded aspartate in the *Escherichia coli* metal transporter YiiP (FieF). *J Biol Chem* **280**, 33716-33724 (2005).

8. Wei Y, Fu D. Binding and transport of metal ions at the dimer interface of the *Escherichia coli* metal transporter YiiP. *J Biol Chem* **281**, 23492-23502 (2006).

9. Guffanti AA, Wei Y, Rood SV, Krulwich TA. An antiport mechanism for a member of the cation diffusion facilitator family: divalent cations efflux in exchange for K+ and H+. *Molecular microbiology* **45**, 145-153 (2002).

10. Beloin C*, et al.* Characterization of an lrp-like (*lrpC*) gene from *Bacillus subtilis*. *Mol Gen Genet* **256**, 63-71 (1997).

11. Ferson AE, Wray LV, Jr., Fisher SH. Expression of the *Bacillus subtilis gabP* gene is regulated independently in response to nitrogen and amino acid availability. *Molecular microbiology* **22**, 693-701 (1996).

12. Ikeda M, Nakagawa S. The *Corynebacterium glutamicum* genome: features and impacts on biotechnological processes. *Appl Microbiol Biotechnol* **62**, 99-109 (2003).

13. Diels L, Dong Q, van der Lelie D, Baeyens W, Mergeay M. The czc operon of

Alcaligenes eutrophus CH34: from resistance mechanism to the removal of heavy metals. *J Ind Microbiol* **14**, 142-153 (1995).

14. Munkelt D, Grass G, Nies DH. The chromosomally encoded cation diffusion facilitator proteins DmeF and FieF from *Wautersia metallidurans CH34* are transporters of broad metal specificity. *Journal of bacteriology* **186**, 8036-8043 (2004).

15. Uebe R*, et al.* The cation diffusion facilitator proteins MamB and MamM of *Magnetospirillum gryphiswaldense* have distinct and complex functions, and are involved in magnetite biomineralization and magnetosome membrane assembly. *Molecular microbiology* **82**, 818-835 (2011).

16. Grover A, Sharma R. Identification and characterization of a major Zn(II) resistance determinant of *Mycobacterium smegmatis*. *Journal of bacteriology* **188**, 7026-7032 (2006).

17. Nies DH. Efflux-mediated heavy metal resistance in prokaryotes. *FEMS Microbiol Rev* **27**, 313-339 (2003).

18. Cubillas C, Vinuesa P, Tabche ML, Garcia-de los Santos A. Phylogenomic analysis of Cation Diffusion Facilitator proteins uncovers Ni2+/Co2+ transporters. *Metallomics : integrated biometal science* **5**, 1634-1643 (2013).

19. Spada S, Pembroke JT, Wall JG. Isolation of a novel *Thermus thermophilus* metal efflux protein that improves *Escherichia coli* growth under stress conditions. *Extremophiles* **6**, 301-308 (2002).

20. Huang DL*, et al.* The Zur of *Xanthomonas campestris* functions as a repressor and an activator of putative zinc homeostasis genes via recognizing two distinct sequences within its target promoters. *Nucleic acids research* **36**, 4295-4309 (2008).

21. Li S, Wang W, Li X, Fan K, Yang K. Genome-wide identification and characterization of reference genes with different transcript abundances for *Streptomyces coelicolor*. *Sci Rep* **5**, 15840 (2015).

22. Jeong Y*, et al.* The dynamic transcriptional and translational landscape of the model antibiotic producer *Streptomyces coelicolor* A3(2). *Nat Commun* **7**, 11605 (2016).

23. Gilston BA*, et al.* Structural and mechanistic basis of zinc regulation across the *E. coli* Zur regulon. *PLoS Biol* **12**, e1001987 (2014).
